# Can a Computer-based Force Feedback Hip Fracture Skills Simulator Improve Clinical Task Performance? A Cadaveric Validation Study

**DOI:** 10.5435/JAAOSGlobal-D-22-00056

**Published:** 2023-05-16

**Authors:** Christopher Domes, Max Coale, Annie Weber, Marckenley Isaac, Ugo Udogwu, Nathan N. O'Hara, Matthew Christian, Robert V. O'Toole, Marcus F. Sciadini

**Affiliations:** From R Adams Cowley Shock Trauma Center, Department of Orthopaedics, University of Maryland Medical School, Baltimore, MD.

## Abstract

This cadaveric study seeks to determine whether skills acquired on the simulator translate to improved performance of the clinical task. We hypothesized that completion of simulator training modules would improve performance of percutaneous hip pinning.

**Methods:**

Eighteen right-handed medical students from two academic institutions were randomized: trained (n = 9) and untrained (n = 9). The trained group completed nine simulator-based modules of increasing difficulty, designed to teach techniques of placing wires in an inverted triangle construct in a valgus-impacted femoral neck fracture. The untrained group had a brief simulator introduction but did not complete the modules. Both groups received a hip fracture lecture, an explanation and pictorial reference of an inverted triangle construct, and instruction on using the wire driver. Participants then placed three 3.2 mm guidewires in cadaveric hips in an inverted triangle construct under fluoroscopy. Wire placement was evaluated with CT at 0.5 mm sections.

**Results:**

The trained group significantly outperformed the untrained group in most parameters (*P* ≤ 0.05).

**Conclusions:**

The results suggest that a force feedback simulation platform with simulated fluoroscopic imaging using an established, increasingly difficult series of motor skills training modules has potential to improve clinical performance and might offer an important adjunct to traditional orthopaedic training.

Percutaneous, fluoroscopically guided surgical procedures are common in fracture surgery, including in the treatment of fractures of the femoral neck, femoral and tibial shaft, and pelvic ring, among others. Accurate and efficient performance of these procedures requires development of specialized surgical skills which include an understanding of the tactile feedback received as a pin traverses various osseous structures. Also required is an ability to process two-dimensional fluoroscopic images and translate them into accurate adjustment of pin trajectories in three dimensions. Simulation training has been shown to be effective in accelerating motor skill acquisition in a risk-free environment for a variety of surgical and nonsurgical applications.^1,2,3,4,5,6,7^ Any new simulation training platform requires validation of its ability to achieve this goal before it can be considered a valuable and effective tool for skill acquisition.

In recognition of the potential value of surgical simulation training as an adjunct to traditional orthopaedic education, a computer-based force feedback simulation platform to teach motor skills associated with percutaneous, fluoroscopically guided procedures was developed. The goal was to facilitate the acquisition of motor skills necessary for the routine performance of fluoroscopically guided orthopaedic procedures. Development of a validated simulation platform for fluoroscopically guided procedures would allow for practice of surgical skills in a nonclinical setting, thereby potentially reducing intraoperative radiation exposure, surgical time, and harm to patients. Once this platform was developed, a three-step process was undertaken to validate the simulation platform used in this study. First, construct validity was demonstrated by showing the ability of the simulator to distinguish between novice and experienced users.^8^ Next, a set of training modules designed to teach motor skills associated with a specific surgical procedure (hip pinning) to improve the performance of this procedure on the simulator was demonstrated.^9^ This study seeks to complete the third step of the process by demonstrating the ability of the simulation platform in conjunction with the training modules to improve performance in the near clinical setting of percutaneous pin placement in cadaver hips.

Our hypothesis was that users who completed the set of training modules would outperform those who did not complete the modules on the task of placing three pins in an inverted triangle configuration in a cadaver under fluoroscopic guidance.

## Methods

After obtaining institutional review board approval, 18 right-handed, first-year and second-year medical students were recruited from two local academic institutions. The participants were block randomized (three blocks of six participants) to either a trained (n = 9) or an untrained (n = 9) group. Trained group participants completed nine simulator-based modules designed to deconstruct the procedure of placing three wires in an inverted triangle construct in a valgus-impacted femoral neck fracture (OTA 31-B1) into basic motor skills components. The training modules then progress through increasing levels of difficulty and complexity culminating in the final assessed task of placing the three wires in the designated configuration. A detailed description of these training modules has been previously published.^2^ The untrained group received introductory training on the use of the simulator including hands-on orientation to familiarize them with it, but participants did not complete the sequential training modules. Within 7 days of randomization and training, participants conducted the procedure of placing pins in whole-body cadaver hips using real fluoroscopic imaging.

**Figure 1:**
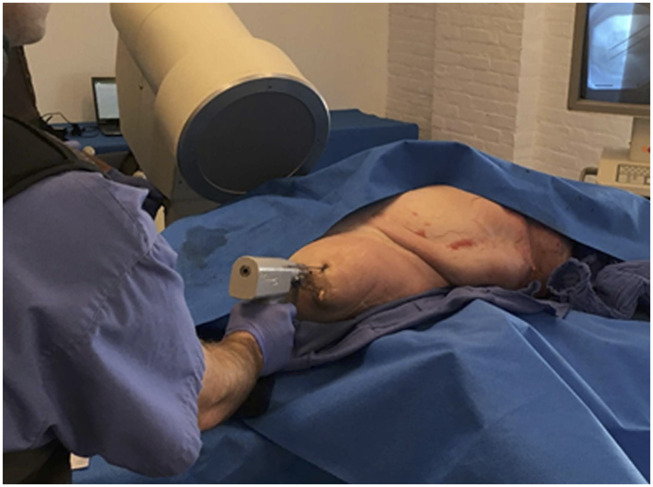
Photograph showing the testing of participant on cadaveric specimens.

On the day of cadaveric testing, both groups received identical short lectures on hip fractures including epidemiology, anatomy, ideal placement, and goals of percutaneous screw fixation. Participants from both groups were given a pictorial reference of an inverted triangle construct to which they could refer during testing, with an AP view and a lateral view of the femoral neck and head. Both groups were instructed on the use of a power driver (Stryker) and were allowed to use the driver on a sawbones model until they felt comfortable with its use. Participants were then shown the standardized C-arm fluoroscopic AP and lateral images of their cadaveric hips. Protective lead aprons were supplied. Participants were then asked to place three 3.2 mm threaded guidewires (Stryker) percutaneously into the hips of supine cadavers under fluoroscopic imaging in the desired inverted triangle construct (Figure 1). They were required to ask for each desired fluoroscopic shot. No limits were placed on the amount of time or number of fluoroscopic images necessary to complete the procedure. Once participants were satisfied with their pin placement, the study was considered completed.

**Figure 2:**
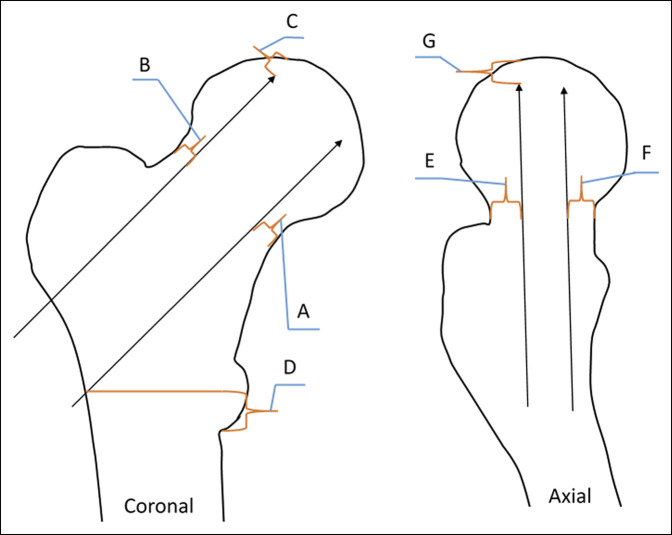
Diagram showing the measurements (in millimeters) obtained with Impax after hip pinning. Coronal view CT measurements: (**A**) distance from pins to inferior mid-neck, (**B**) distance from pins to superior mid-neck, (**C**) distance from pins to articular surface, and (**D**) distance from bottom of lesser trochanter to most inferior pin. Axial view CT measurements: (**E**) distance from pin to anterior mid-neck, (**F**) distance from pin to posterior mid-neck, and (**G**) distance from end of pin to femoral head.

**Figure 3:**
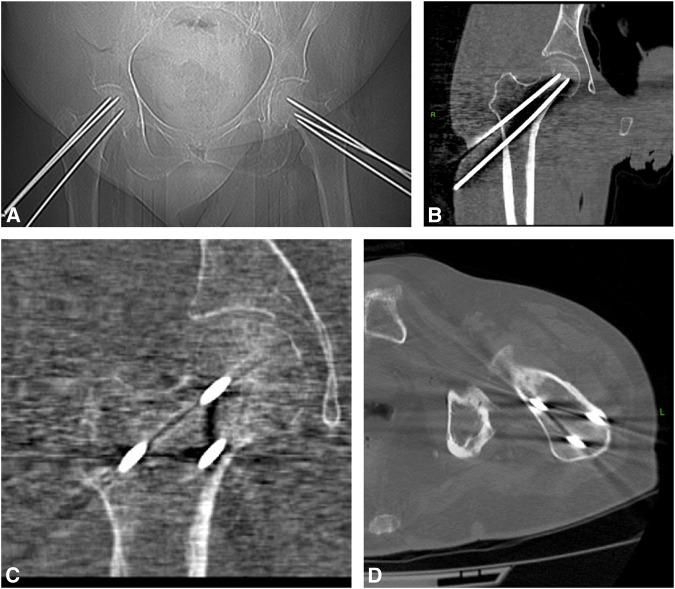
Diagram showing (**A**) CT anterior-posterior scout image. **B** and **C**, Coronal view sections show wires in the femoral neck. **D**, Axial view section shows wires in the femoral neck.

Wire placement in the cadaveric specimens was evaluated with CT at 0.5 mm sections. Impax (AFGA) was used to evaluate the acquired images. CT data were collected by the primary investigator, who was blinded to the status of the participants at the time of data collection and analysis. Fluoroscopic time, number of AP and lateral view images, and apparent femoral head penetrations were recorded. Performance was assessed based on outcome measures determined from previous construct validation studies,^1,2^ including pin distance to ideals for the middle inferior, superior anterior, and superior posterior aspects of the femoral neck, and to the articular surface of the femoral head and distance from the lower border of the lesser trochanter to the most inferior pin (Figure 2). An assessment of whether an inverted triangle construct was achieved was made by the primary investigator by evaluating the postprocedure CT scans and final AP and lateral fluoroscopic images (Figure 3).

Performance metrics were compared between the two groups using a Student *t*-test for continuous variables and a Fisher exact test for binary variables. All analyses were done using JMP Pro Version 13 (SAS Institute).

## Results

The trained group was significantly better at achieving an inverted triangle construct compared with the untrained group (8 of 9 [89%] versus 3 of 9 [33%], *P* = 0.05). No significant differences were observed between the trained and untrained groups regarding either the number of AP or lateral view images obtained or total fluoroscopic time (Table 1).

**Table 1:** Radiographs Obtained and Fluoroscopic Time per Group

Fluoroscopic Parameters	Trained Group (n = 9)	Untrained Group (n = 9)	
	Mean (SD)		*P* Value
Anterior-posterior view radiographs, n	47.6 (23.4)	32.1 (27.2)	0.22
Lateral view radiographs, n	31.4 (44.1)	17.4 (20.5)	0.41
Fluoroscopic time, s	47.4 (38.8)	29.7 (28.0)	0.29

No significant differences were found between the trained and untrained groups regarding distance from the tip of the wire to the femoral head articular surface as shown by CT in the axial and coronal planes. Overall, the trained group did trend toward being closer to this surface than the untrained group. This was especially true for the superior posterior pin (Table 2).

**Table 2:** Distance From Pin Tip to Femoral Head

Pin Parameters	Trained Group (n = 9)	Untrained Group (n = 9)	
	Mean mm (SD)		*P* Value
Axial: Superior posterior pin	22.8 (11.0)	34.2 (12.8)	0.06
Coronal: Superior posterior pin	13.9 (8.1)	22.1 (13.5)	0.14
Axial: Superior anterior pin	22.0 (8.0)	23.1 (6.8)	0.75
Coronal: Superior anterior pin	16.3 (7.3)	24.4 (10.2)	0.07
Axial: Inferior/calcar pin	24.4 (15.9)	26.8 (10.6)	0.72
Coronal: Inferior/calcar pin	16.4 (11.8)	22.6 (16.2)	0.37

Significant differences in performance metrics as measured from the CT scans were found to favor the trained group participants in several parameters for all three pins in the inverted triangle construct. The results, shown in Table 3, showed significant differences in assessed parameters for proper pin placement, including distance to the mid-cervical cortex for all three pins, distance from the ideal lateral starting point for the inferior pin, and parallel placement of the pins in relation to each other.

**Table 3:** Distance From Pin to Designated Location and Degrees Between Corresponding Pins

Pin Parameters	Trained Group (n = 9)	Untrained Group (n = 9)	
	Mean (SD)		*P* Value
Angle between inferior and superior posterior pin, degree	2.8 (2.1)	6.3 (2.0)	<0.01
Angle between inferior and superior anterior pin, degree	3.0 (2.2)	6.1 (2.8)	0.02
Angle between superior posterior and superior anterior pin, degree	3.3 (2.8)	5.9 (2.3)	0.05
Superior posterior pin distance to mid-neck, mm	7.5 (4.9)	16.2 (10.7)	0.05
Superior anterior pin distance to mid-neck, mm	6.2 (3.0)	13.8 (5.3)	<0.01
Inferior/calcar pin distance to calcar mid-neck, mm	8.2 (6.6)	14.1 (5.1)	0.05
Distance above bottom of lesser trochanter, mm	17.8 (8.3)	28.0 (11.6)	0.05

## Discussion

Surgical training has relied on the mentorship model first put forth by Halsted in 1904.^10^ In that model, a trainee is taught surgical skills by observing, assisting, and eventually performing procedures in the operating room under the supervision of a more experienced surgeon mentor. In theory, increasing autonomy in the operating room comes as the trainee's experience, knowledge, and skills improve. Eventually, the trainee is deemed by the surgeon mentor to be competent to independently perform surgical procedures. This training methodology is, on the surface, based on a goal-driven educational paradigm. However, because of multiple factors, including limited time in the operating room, limited exposure to certain surgical procedures over the course of a surgical training program, and trainee expectations of increased autonomy based on year in training, this system is prone to devolving into a time-driven educational paradigm. In addition, mentor assessment of trainee proficiency is highly susceptible to subjectivity and potential bias. Furthermore, hands-on learning of complex surgical procedures has the potential to subject patients to increased intraoperative risk based on increased surgical times and risk of technical errors.

The major accreditation council for American residencies, the Accreditation Council for Graduate Medical Education, has implemented several quality control measures to assure that surgical residents in the United States have a baseline level of technical experience and proficiency in addition to an appropriate amount of clinical duty. These interventions have included the addition of case minimums, duty hour restrictions, and yearly resident surveys regarding their program.^11^ Varying levels of support for these measures have been reported by both surgical residents and their program administrators.^12,13,14,15^ The coding accuracy, which is how minimums are captured, has been called into question, and high levels of variance in accuracy of reporting have been noted for surgical residents and orthopaedic fellows.^15,16^ These data suggest that although minimums can help provide some baseline level of surgical experience and exposure to critical procedures, reporting accuracy is suspect; furthermore, merely tracking and recording resident case logs do not guarantee competency in the procedures.

Advances in technology and recognition of the utility of surgical simulation provide additional opportunities to intervene and supplement surgical training. Simulation training might be more appropriate and effective in skill development and assessment for some surgical procedures than it is for others. Skills training through simulation has been shown to be effective for laparoscopic and arthroscopic procedures, and robust simulation training modalities and platforms are well established for those procedures.^17,18,19,20,21^ Early development of simulation training in laparoscopy was possible in large part because of the different skill set required for laparoscopic procedures compared with traditional open surgery, and laparoscopic surgical simulation has become a requisite component of general surgical residency training.^18,19^ Within orthopaedics, arthroscopic surgery has been done for more than 50 years and predates laparoscopy. Although arthroscopy shares many of the requisite motor skills with laparoscopy, its long-standing presence in the realm of orthopaedic surgery means it was taught for decades using the traditional surgical training paradigm. Only recently has the concept of simulation training been applied to arthroscopy.^5–7,22,23^ Arthroscopic simulation training platforms currently available include complex computer-based simulators, foam models, and simple box models for basic skills development, several of which have been validated in previous studies.^5–7^

Percutaneous fluoroscopically guided procedures are very commonly done to treat adult and pediatric trauma and are ideal for the design and implementation of equally robust simulation training platforms to augment traditional approaches for the development of requisite motor skills and understanding of instrumenting three-dimensional anatomic structures using two-dimensional imaging. Simulators and associated training exercises can be designed to provide trainees the opportunity to practice and improve visual motor skill acquisition in an environment that is safe and nonthreatening to both trainees and patients and have the potential to provide accurate real-time assessment of performance and simulated procedural mastery.

The simulation platform used in this study offers all these desired features. Training modules were developed to teach the fundamentals of percutaneous pinning of a valgus-impacted femoral neck fracture.^8,9^ This model was deemed to be appropriate for initial development and validation of the simulator because of its relative simplicity and consistency. Considering that nearly 300,000 hip fractures occur in the United States each year, treatment of these fractures is a commonly performed surgical procedure for both trainees and practicing surgeons and ability to perform this procedure is considered a core competency by the Accreditation Council for Graduate Medical Education. The basic motor skills necessary for performance of percutaneous hip pinning also provide the foundation for more complex percutaneous fluoroscopically guided procedures. Although a relatively simple procedure, percutaneous pinning of femoral neck fractures requires notable hand-eye coordination and visual-spatial awareness in addition to effective use of fluoroscopic guidance.^8,9,24–26^ Development of these surgical skills through the traditional mentorship model likely leads to increases in both surgical and fluoroscopic time and can affect patient well-being. In 2010, Bjorgul et al^26^ showed that a learning curve exists, as indicated by mean surgical time, for residents performing hip fracture pinning. The authors found that surgical times improved from 47.8 minutes at initiation to 30.1 minutes for procedures numbered 21 through 25.

The fluoroscopically guided procedure simulator in this study has been previously validated in its ability to distinguish between novice and experienced practitioners.^8^ An additional validation study noted efficacy of the motor skills training modules in improving trainee performance on the simulated placement of three pins in an inverted triangle construct in the femoral neck, as determined by metrics measured on and by the simulator itself.^9^ The goal of this study was to determine whether training on the simulator and completion of the skills modules designed for percutaneous hip pinning would confer transfer of surgical skills to the actual clinical procedure, mimicked by performance of the procedure on a cadaver.

Our study showed that after training with the simulator, participants were better able to place the guidewires in the desired inverted triangle construct in a human cadaver. Trained participants were more accurate with the placement of their guide pins in relation to the ideal position at the mid-femoral neck and with the placement of the pins parallel to each other.

Tremendous potential educational benefits are associated with transitioning some of the initial training and motor skills acquisition from the surgical theater on live patients to the simulated surgical setting. Factors including enhanced patient safety, learning the judicious use of fluoroscopy, improved understanding of basic surgical principles, development of motor skills, and proper interpretation of tactile feedback can all be affected positively by the incorporation of an effective fluoroscopic simulation platform. Interpersonal factors, such as increased surgical confidence, can also be influenced by providing trainees with the opportunity to develop surgical skills in an independent, nonthreatening environment where the principles of goal-driven learning can be more effectively applied. The importance of these potential gains cannot be overstated. Factors such as duty hour restrictions and resultant decreased time in the operating room for trainees conflict with other factors such as increased prevalence of percutaneous surgical techniques and reliance on intraoperative fluoroscopy for the treatment of common fractures.

This study should be interpreted within the context of its limitations. It was done with volunteer, right-handed medical students who, at this stage of their careers, are not yet committed to pursuing surgical training. There could be inherent differences between general medical students and those who decide to go into a hands-on surgical field, conceivably yielding different results with a different group of participants. The use of medical students for this validation study, as opposed to orthopaedic surgical trainees, was deliberate. The goal was to evaluate “clean slate” learners as much as possible, with minimal if any previous exposure to or experience with percutaneous surgical procedures. Future studies to determine the usefulness and effectiveness of this simulation platform are planned and intend to focus on orthopaedic surgical trainees. There were nine participants per group, which was deemed adequate by the power analysis based on the previous validation study.^8,9^ However, applying this study protocol to a larger group of volunteers might yield different results. Our methodology differs from that established in previous validation studies for arthroscopy simulation^5–7^ primarily in that the assessment of the efficacy of simulation training was based on the surgical procedure being performed and evaluated in cadavers rather than live patients. Although this difference might be perceived as a weakness of this study by proponents of the established validation protocol for arthroscopy, it could also be considered a strength based on the following considerations: (1) our protocol removes the variable associated with heterogeneity of fracture patterns inherent to attempting to quantify performance on actual patients, (2) use of cadavers allowed for participants in the trained group to be evaluated in a closely controlled time frame in relation to their simulation training, and (3) objective evaluation of pin placement through direct measurement from CT is arguably more accurate than subjective evaluation of a learner's performance in the clinical setting by a surgical mentor. A more accurate approximation of the clinical scenario could have included a surgeon mentor assisting and taking the participants in both groups through the procedure on the cadaver. This was not done in this study but might have yielded different results.

Although no statistical differences in fluoroscopic time or number of images taken to complete the task were found between groups, the trained participants had higher values for all of these parameters. This finding could reflect a better understanding of the task and a higher level of awareness of the importance of correct pin placement by virtue of having completed the training modules but runs counter to the stated goal of decreasing intraoperative fluoroscopic exposure. This study did not determine the amount of fluoroscopic time required by either group to attain what would be considered “acceptable” pin placement. Having conducted this study as described above, with a surgeon mentor assisting with the procedure, would have helped define any effect of simulation training on satisfactory completion of the task.

Based on the results of this study, it seems that this fluoroscopic simulator and the currently developed series of training modules provide a valuable adjunct to traditional surgical training methodology for teaching the surgical skill of percutaneous pinning of femoral neck fractures. It is possible that by applying a similar approach to the stepwise development and validation of simulated procedures and associated training modules to additional fluoroscopically guided surgical procedures, this simulation platform could offer a wide-ranging educational value. Additional study is necessary to determine the applicability of this methodology to additional fluoroscopic procedures, simulator-acquired skill retention over time, and translation of skill acquisition to actual improved performance in the clinical setting.

## Conclusion

This study demonstrates potential transfer of skill from a computer-based, force feedback fluoroscopy simulator to clinical performance, mimicked here by human cadaver surgery. It reinforces the utility of this simulator in training future practitioners on the pinning of femoral necks. Furthermore, the development of additional training modules for more complex surgical procedures would increase the overall utility of this simulator in orthopaedic training (Supplemental Information, http://links.lww.com/JG9/A282).
